# Absence of Plekhg5 Results in Myelin Infoldings Corresponding to an Impaired Schwann Cell Autophagy, and a Reduced T-Cell Infiltration Into Peripheral Nerves

**DOI:** 10.3389/fncel.2020.00185

**Published:** 2020-07-07

**Authors:** Patrick Lüningschrör, Carsten Slotta, Peter Heimann, Michael Briese, Ulrich M. Weikert, Bita Massih, Silke Appenzeller, Michael Sendtner, Christian Kaltschmidt, Barbara Kaltschmidt

**Affiliations:** ^1^Institute of Clinical Neurobiology, University Hospital Wuerzburg, Wuerzburg, Germany; ^2^Department of Cell Biology, University of Bielefeld, Bielefeld, Germany; ^3^Molecular Neurobiology, University of Bielefeld, Bielefeld, Germany; ^4^Core Unit Systems Medicine, University of Wuerzburg, Wuerzburg, Germany; ^5^Comprehensive Cancer Center Mainfranken, University Hospital Wuerzburg, Wuerzburg, Germany

**Keywords:** Schwann cells, autophagy, immune response, myelin, PLEKHG5

## Abstract

Inflammation and dysregulation of the immune system are hallmarks of several neurodegenerative diseases. An activated immune response is considered to be the cause of myelin breakdown in demyelinating disorders. In the peripheral nervous system (PNS), myelin can be degraded in an autophagy-dependent manner directly by Schwann cells or by macrophages, which are modulated by T-lymphocytes. Here, we show that the NF-κB activator Pleckstrin homology containing family member 5 (Plekhg5) is involved in the regulation of both Schwann cell autophagy and recruitment of T-lymphocytes in peripheral nerves during motoneuron disease. *Plekhg5*-deficient mice show defective axon/Schwann cell units characterized by myelin infoldings in peripheral nerves. Even at late stages, *Plekhg5*-deficient mice do not show any signs of demyelination and inflammation. Using RNAseq, we identified a transcriptional signature for an impaired immune response in sciatic nerves, which manifested in a reduced number of CD4^+^ and CD8^+^ T-cells. These findings identify Plekhg5 as a promising target to impede myelin breakdown in demyelinating PNS disorders.

## Introduction

The myelin sheath is an essential cellular component for axonal integrity and function. Although oligodendrocytes generate myelin in the central nervous system (CNS), in the peripheral nervous system (PNS) it is formed by Schwann cells (Nave and Werner, 2014). Nerve conduction velocity (NCV) is highly dependent on an intact myelin sheath. However, in pathological situations or in the context of neurodegenerative diseases, such as hereditary motor and sensory neuropathies (HMSN), myelin breakdown (demyelination) can occur, leading to impairments in axonal integrity and function and also to axonal loss. At the same time, several demyelinating neuropathies are associated with excessive myelin production that precedes demyelination and axon loss. Histologically, this appears as myelin outfoldings or focal thickening of the myelin sheath (tomacula; Quattrone et al., 1996; Fabrizi et al., 2000; Bolino et al., 2004; Lee et al., 2013).

Abnormalities of the myelin architecture, especially in the context of neurodegenerative diseases, are often linked to inflammation and an altered immune response. By suppressing the immune response, myelin maintenance can be improved in mouse models for a demyelinating CNS disorder (Ip et al., 2006) and Charcot Marie Tooth (CMT) disease (Schmid et al., 2000). Although myelin breakdown in the CNS is mediated predominantly by macrophages (Brosius Lutz and Barres, 2014), within the PNS, Schwann cells degrade their myelin sheath *via* a specific form of autophagy (myelinophagy) in response to axonal stress (Gomez-Sanchez et al., 2015).

The guanine exchange factor Plekhg5 (also known as Syx or Tech) is a known activator of NF-κB (Maystadt et al., 2007), which is highly expressed within the nervous system (De Toledo et al., 2001; Marx et al., 2005). Mutations within the human *PLEKHG5* gene are associated with different motoneuron diseases, such as an intermediate form of CMT, distal spinal muscular atrophy (DSMA) type IV, and amyotrophic lateral sclerosis (ALS; Maystadt et al., 2007; Azzedine et al., 2013; Kim et al., 2013; Özoğuz et al., 2015). Mice lacking Plekhg5 developed a late-onset motoneuron disease caused by impaired autophagy-mediated clearance of synaptic vesicles at the neuromuscular junctions (Lüningschrör et al., 2017). However, the peripheral nerves of Plekhg5-deficient mice have not been investigated yet.

In this study, we histologically examine the peripheral nerves of Plekhg5-deficient mice and detect myelin abnormalities characterized by infolding of the myelin sheath. We report an impaired myelin clearance by Schwann cell autophagy and a reduced macrophage activity in cultured nerve segments derived from Plekhg5-depleted mice. Using RNA-sequencing, we find a prominent downregulation of macrophage transcripts, including a number of chemokines for T-cell attraction. In line with that, we observe a reduced number of T-lymphocytes within the sciatic nerve indicating an impaired immune response despite axonal pathology.

## Materials and Methods

### Statistical Analysis

Statistical evaluation was done using GraphPad Prism 5 (GraphPad Software, La Jolla, CA, USA). Data is presented as the mean ± SEM if not stated otherwise. The statistical test used for each experiment is listed within the respective figure legend.

### Animals

*Plekhg5*-deficient mice were described previously (Lüningschrör et al., 2017). Animals were kept under specific pathogen-free conditions as defined by the Federation European Laboratory Animal Science Association (FELASA) in the central animal facility of the University of Bielefeld.

### Light and Electron Microscopy

Mice were anesthetized and transcardially perfused according to local institutional guidelines in three steps essentially as described by Forssmann et al. (1977) with 3% paraformaldehyde, 3% glutaraldehyde, 0.5% picric acid in 0.1 M sodium phosphate buffer, pH 7.4 for 10 min. After dissection, organs were fixed in the same solution for additional 2 h at 4°C followed by 2 h post-fixation in buffered 2% osmium tetroxide at 4°C. Afterward, they were embedded in Araldite. For light microscopy, semithin sections were stained with Richardson’s blue (1% w/v methylene blue, 1% w/v Azur II) for 3 min, 80°C. For electron microscopy using a Zeiss EM 109, ultra-thin (60–80 nm) sections (stained for 40 min in uranyl acetate and 7 min in lead citrate) were prepared.

### Morphometric Analysis and Determination of g-Ratio

Nerve fibers with infolded myelin membranes or internal myelin loops were defined as fibers with abnormal myelination. The percentage of fibers with abnormal myelination was quantified on semithin sections. All myelinated axons per section were used for quantification.

Semithin sections were used for analyzing the g-ratio of myelinated axons within the sciatic nerve. High-resolution images were taken and afterward stitched together with Photoshop CS software (Adobe Systems) resulting in a high-resolution image of the whole sciatic nerve cross-section. Using the g-ratio plugin for ImageJ software (Goebbels et al., 2012), 100 randomly chosen axons per cross-section were measured. Fibers with myelin abnormalities were excluded.

### Immunohistochemical Stainings

Mice were sacrificed by cervical dislocation, the sciatic nerve was dissected, embedded in Tissue-Tec O.C.T. compound (Sakura Finetek) and frozen in 2-methylbutane at −30°C. Then, 10-μm thick sections were cut using a CM1900 microtome (Leica Microsystems). Frozen sections were shortly thawed and, depending on primary antibody, they were fixed using −20°C cold acetone for 10 min, with 4% paraformaldehyde for 10 min or left unfixed. Following repetitive washing using phosphate-buffered saline (PBS), sections were blocked in PBS containing 5% BSA. Primary antibodies were applied overnight at 4°C, followed by washing in PBS and application of secondary fluorochrome-conjugated antibodies. For nuclear counterstaining DAPI (1 μg/ml; AppliChem) was used. After washing, sections were finally coverslipped with Mowiol/DABCO.

For stainings of cultivated nerve segments, segments were fixed in 4% PFA for 1 h, followed by repetitive washing. Teased fiber preparations were done using fine forceps. Fibers were placed on collagen-coated coverslips, and after permeabilization using 0.3% Triton X-100, staining was performed as stated above.

### RNA Extraction and qPCR

For extraction of total RNA, TRIReagent (Sigma-Aldrich) was used according to the manufacturer’s protocol. Then, 500 ng of RNA were used for cDNA synthesis. cDNA was diluted 1:50 and 2 μl/reaction were used. qPCR was carried out with SYBR Green Master Mix (Thermo Fisher Scientific). For normalization, we used the mean expression of the two housekeeping genes *Ppia* (Cyclophilin A) and *Eef2* (Eukaryotic elongation factor 2).

### Lentivirus Production

For lentivirus production, HEK 293FT cells were transfected with the plasmids indicated and packaging plasmids VSV-G and delta8.91 using standard calcium phosphate precipitation. Eight hours after transfection, medium was exchanged, and 48–72 h after transfection, supernatants were collected and concentrated by ultracentrifugation. For transduction, lentiviral particles were diluted in the respective growth medium, and polybrene was added to a final concentration of 8 μg/ml.

### Primary Schwann Cell Culture

Murine neonatal Schwann cells were cultured essentially as described (Honkanen et al., 2007). Neonatal animals were sacrificed by decapitation at postnatal days 5 and 6. The sciatic nerves were dissected and maintained in ice-cold PBS until all nerves were prepared. Remaining connective tissue was removed, and the nerves were transferred to a new dish containing fresh ice-cold PBS, where they were shredded with forceps. Following enzymatic digestion using trypsin (final concentration 0.125%) and collagenase A (final concentration 0.05%) for 30 min at 37°C, nerve fragments were centrifuged for 5 min at 190× *g*. After three washing steps with 7 ml DMEM containing 10% horse serum and centrifugation for 5 min at 190× *g*, the pellet was resuspended in basic growth medium (DMEM containing 10% horse serum, 4 mM L-Glutamine, 100 μg/ml Penicillin/Streptomycin, 2 ng/ml human heregulin-β1 and 0.5 μM forskolin), plated on a poly-D-lysine–coated 60-mm tissue culture dish and incubated at 37°C and 5% CO_2_. After 2 days in culture, basic growth medium was replaced, and the cells were allowed to grow for two additional days. To remove fibroblasts, complement-mediated cytolysis was done at day 4 in culture. Medium was removed, and the cells were rinsed with HBSS in 20 mM HEPES, followed by rinsing with HMEM (DMEM containing 10% horse serum, 4 mM L-glutamine, 100 μg/ml penicillin/streptomycin, and 20 mM HEPES). Antimouse CD90 antibody was diluted in HMEM to a final concentration of 4 μg/ml and added to the cells. After 15 min at 37°C, complement sera was added, and incubation continued for an additional 2 h. Cells were rinsed twice with HBSS containing 20 mM HEPES, and finally, Schwann cell growth medium (basic growth medium containing 10 ng/ml FGF-2 and 20 μg/ml bovine pituitary extract) was added. Medium was changed every 2 days, and cells were subcultured when reaching 80% confluence. All cells were passaged at least once before being used for experiments.

### RNAseq

Total RNA was isolated from sciatic nerves using TRIReagent (Sigma-Aldrich) according to the manufacturer’s guidelines, and cDNA library generation was performed using the SENSE mRNA-Seq Library Prep Kit V2 (Lexogen, Vienna, Austria) according to the manufacturer’s protocol. Libraries were pooled and sequenced on the Illumina NextSeq 500 with the High Output Kit v2 (75 cycles). Adapters and low-quality reads were trimmed with TrimGalore, v0.4.0^1^ powered by Cutadapt, v1.8^2^ (Martin, 2011). Additionally, the first nine nucleotides were removed as described in the Lexogen user manual. Reads were then mapped using STAR v2.5.0a^3^, and differentially expressed transcripts were determined using the Cufflinks package v2.2.1^4^ as described before (Briese et al., 2016). Venn diagrams were generated using BioVenn (Hulsen et al., 2008). For gene ontology (GO) analysis, we used the Database for Annotation, Visualization, and Integrated Discovery (DAVID^5^; Huang et al., 2009). As a background data set for the GO term analysis, we used all expressed genes, which we defined as those transcripts with an average FPKM ≥0.1 in either the *Plekhg5*-deficient or wild-type sciatic nerve data sets. NF-κB targets were extracted from https://www.bu.edu/nf-kb/gene-resources/target-genes/. The sequencing files have been deposited in NCBI’s Gene Expression Omnibus (Edgar et al., 2002) and are accessible through GEO Series accession number GSE127319.

### Cultivation of Sciatic Nerve Segments

Sciatic nerve segments were cultivated as described (Gomez-Sanchez et al., 2015). Briefly, adult mice were sacrificed by cervical dislocation, and the sciatic nerves were prepared and placed on ice in HBSS. Connective tissue was removed and the desheathed nerves were cut into 5-mm-long segments. Segments were transferred to culture medium (DMEM containing 5% FCS) and cultivated for 5 days at 37°C and 5% CO_2_.

To quantify the number of intact myelin sheaths, at least five sections per animal were analyzed. Axons completely surrounded by a myelin sheath were considered as “intact myelin sheath.”

### Quantification of Autophagosomes and Autolysosomes

To monitor the autophagic flux in cultured Schwann cells, the cells were transduced with a lentiviral vector expressing mRFP-GFP-LC3. This tandem reporter is a well-established tool enabling the discrimination between autophagosomes and autolysosomes (Klionsky et al., 2016; Lüningschrör et al., 2017, 2020). Upon fusion of an autophagosome with a lysosome, the GFP signal is quenched due to the pH drop, whereas the RFP signal remains. Thus, structures positive for RFP are autolysosomes, whereas organelles positive for both, GFP and RFP, are considered as autophagosomes.

For quantification of RFP^+^ structures or RFP and GFP double-positive structures, cultured Schwann cells were fixed for 10′ at RT with 4% PFA and mounted on coverslips. Images were evaluated using ImageJ.

## Results

### *Plekhg5*-Deficiency Disrupts the Integrity of Schwann Cell–Axon Units

**Figure 1:**
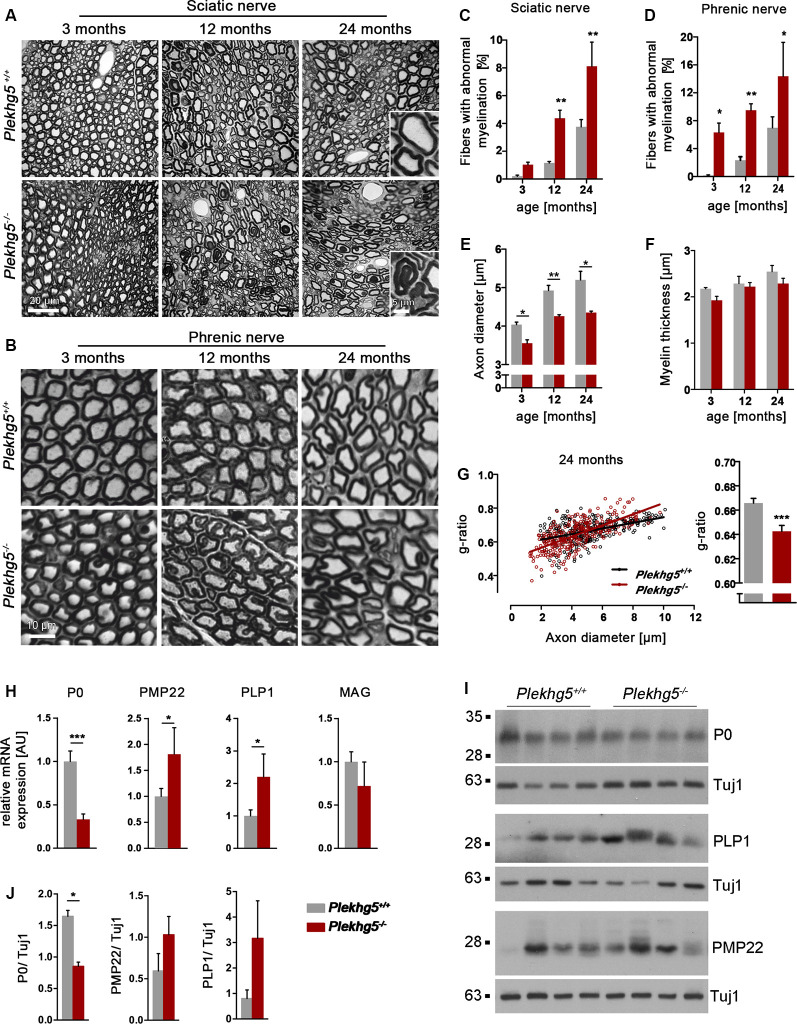
Histopathological analysis of peripheral nerves. **(A,B)** Semithin sections of the sciatic **(A)** and phrenic **(B)** nerve nerve of wild-type (*Plekhg5*^+/+^) and mutant mice (*Plekhg5*^−/−^) at different ages. Note numerous myelination defects in mutant mice. Scale bars: **(A)** 20 μm and 5 μm (blow-up), **(B)** 5 μm. **(C,D)** Relative amount of axons with myelin alterations. Five animals per genotype and age (*n* = 5, unpaired, two-tailed *t-test*). **(E)** Mean axonal diameter of wild-type and mutant mice at different ages. Three animals per genotype and age (*n* = 3, unpaired, two-tailed *t-test*), 100 axons/animal. **(F)** Mean myelin thickness of wild-type and mutant mice at different ages. Three animals per genotype and age (*n* = 3, unpaired, two-tailed *t-test*), 100 axons/animal. **(G)** G-ratio analysis of 24-month-old mice. Three animals per age and genotype, 100 axons/animal. **(H)** qPCR analysis of several myelin genes within the sciatic nerve of wild-type and mutant mice at an age of 10 months [*n* = 5/6 (wild-type/ko), unpaired, two-tailed *t-test*]. The mean expression of the two housekeeping genes Ppia and Eef2 was used as internal control. **(I)** Protein levels of P0, PMP22, and PLP1 in sciatic nerve lysates obtained from 11-month-old mice. **(J)** Quantification of Western blots [*n* = 5/4 (wild-type/ko), unpaired, two-tailed *t-test*]. *p < 0.05; **p < 0.01; ***p < 0.001.

To investigate the effect of Plekhg5 depletion on peripheral nerves, we histologically analyzed the sciatic and phrenic nerve of *Plekhg5*-deficient mice. At 3 months of age, we detected no obvious abnormality in the myelin structure within the sciatic nerve of Plekhg5-deficient mice. However, in the sciatic nerves of 12-month-old Plekhg5-deficient mice, we detected abnormal Schwann cell–axon units with infolded myelin sheaths (Figures 1A,C). In contrast, we already detected a significantly increased number of myelin infoldings in the phrenic nerve of Plekhg5-deficient mice at 3 months of age (Figures 1B,D). In both, the sciatic and phrenic nerves, the number of axons with myelin infoldings, progressed from 12 to 24 months of age (Figures 1A–D). The infolded myelin membranes were either coiled into the axon or appeared as a single internal myelin ring within a myelinated axon (Figures 1A,B). Morphometric analysis revealed a significantly reduced axonal diameter in *Plekhg5*-deficient animals already at an age of 3 months. This reduction remained consistent in 12- and 24-month-old animals (Figure 1E). In contrast, the myelin thickness was unchanged in Plekhg5-deficient mice (Figure 1F). As a direct consequence of the reduced axon diameter, we also detected a reduced g-ratio in 24-month-old animals (Figure 1G). The histological analyses did not show any obvious signs of demyelination and remyelination, such as thinly myelinated large axons or onion-bulb formation in peripheral nerves.

Next, we analyzed the expression of the myelin genes *P0*, *PMP22*, and *PLP1* by qPCR and Western blot (Figures 1H–J). On the transcriptional level, we detected a marked reduction in the expression of *P0* but elevated levels of *PMP22* and *PLP1* (Figure 1H). On the protein level, we only detected a significant decrease in the expression of P0 although we also observed an increase in the levels of PLP1 and PMP22, which did not reach statistical significance (Figures 1I,J).

**Figure 2:**
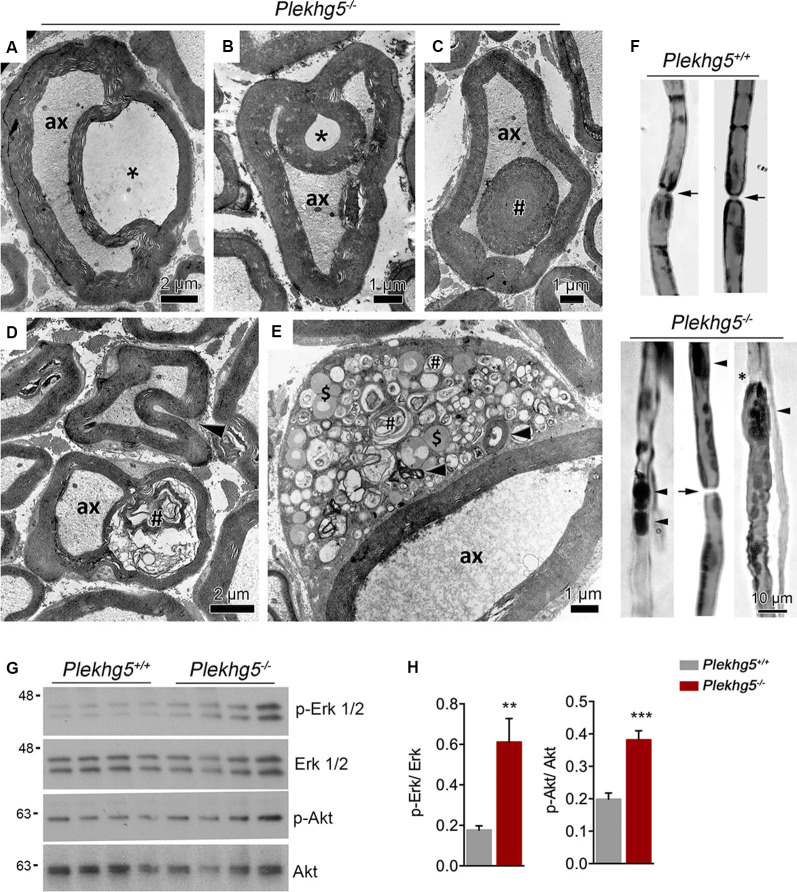
Ultrastructural analysis of sciatic nerve. **(A–C)** Electron micrographs of *Plekhg5*-deficient mice depicting severe pathological features as splitting of myelin sheath **(A)**, myelin infoldings **(B,D)**, and an internal myelin ring within the myelinated axon (# in **C**). Asterisk highlights the lack of axonal or cytosolic structures (e.g., neurofilaments, organelles, etc.) within myelin infoldings. ax = axon; scale bar: 2 μm **(A,D)**, 1 μm **(B,C,E)**. **(D)** Partly disrupted myelin sheath (#) containing myelin debris; note beginning infolding myelin sheath (arrowhead). **(E)** Schwann cell soma with myelin debris (arrowheads), accumulations of vesicles from putatively the autophagosomal (#), and endolysosomal ($) compartment. **(F)** Teased nerve fiber preparation, lipids visualized (=dark staining) by treatment with osmium tetroxide. Arrows point to node of Ranvier, arrowheads to myelin accumulations, note irregular outline in **(E)** and apparent blunt nerve ending (*). Scale bar: 10 μm.** (G)** Western blot analysis of Erk1/2 and Akt signaling in sciatic nerve lysates obtained from 11-month-old mice. **(H)** Quantification of Western blots [*n* = 5/4 (wild-type/ko), unpaired, two-tailed *t-test*]. **p < 0.01; ***p < 0.001.

On the ultrastructural level, infolded myelin sheaths appeared in different forms, most likely representing different infolding stages (Figures 2A–C). In early stages, the infolding myelin membranes were coiled into the axon (Figures 2A,B) before appearing as a single internal myelin ring within a myelinated axon (Figure 2C). Myelin infoldings did not contain axonal or cytosolic structures (Figures 2A,B). We also detected myelin infoldings with accumulations of myelin debris (Figure 2D) and less frequently Schwann cell soma with accumulations of vesicles from the autophagosomal and lysosomal compartment (Figure 2E). Teased fiber preparations showed that myelin abnormalities predominantly originated between paranodal regions (Figure 2F).

Impaired myelination, such as myelin outfoldings, infoldings, or tomacula, have been linked to enhanced activation of Erk1/2 and/or Akt signaling (Goebbels et al., 2012; Lee et al., 2012; Napoli et al., 2012). Western blot analysis of sciatic nerve lysates from Plekhg5-deficient mice revealed elevated levels of both activated Erk1/2 and Akt (Figures 2G,H).

In summary, these results suggest that the depletion of Plekhg5 results in defective Schwann cell axon units characterized by infoldings of the myelin sheath, an altered expression of myelin proteins, and enhanced Erk1/2 and Akt signaling.

### Impaired Formation of Autophagosomes in *Plekhg5*-Deficient Schwann Cells

**Figure 3:**
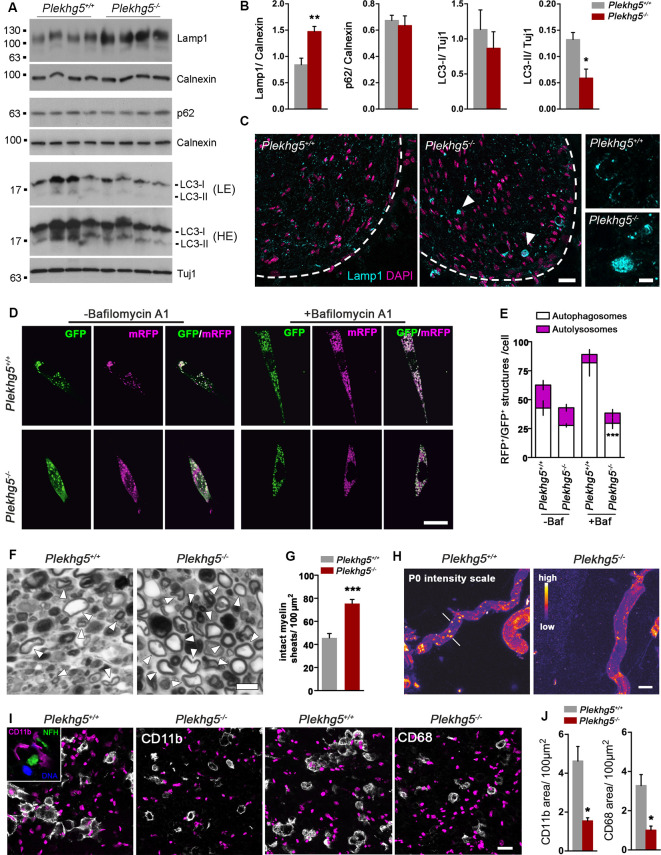
Impairments in Schwann cell autophagy upon *Plekhg5*-deficiency. **(A)** Protein levels of Lamp1, p62, and LC3 in sciatic nerve lysates obtained from 11-month-old mice. LE, low exposure, HE, high exposure. **(B)** Quantification of Western blots (*n* = 5/4 (wild-type/ko), unpaired, two-tailed *t-test*). **(C)** Immunohistochemical staining of sciatic nerve cross-section from 12-month-old animals for Lamp1. Overview; scale bar: 50 μm. Higher magnification; scale bar: 5 μm. Arrowheads point to accumulations of Lamp1^+^ organelles. **(D)** Confocal microscopic analysis of primary Schwann cells transduced with mRFP-GFP-LC3 reporter. To inhibit fusion of autophagosomes with lysosomes, the cells were treated with 400 nM Bafilomycin A1 for 4 h or left untreated prior to fixation. **(E)** Quantitative analysis of fluorescent structures within transduced Schwann cells. GFP^+^–RFP^+^ structures were defined as autophagosomes, GFP^−^–RFP^+^ structures as autolysosomes. At least five cells per experiment were analyzed; three independent experiments (*n* = 3, two-way ANOVA with Bonferroni posttest). **(F)** Representative images of semithin sections of sciatic nerve fragments from wild-type and mutant mice cultivated for 5 days. Arrowheads point to intact myelin sheets. **(G)** Amount of intact myelin sheaths, normalized to the area of the section analyzed. At least five sections per animal were analyzed; three animals per genotype (*n* = 3, unpaired, two-tailed *t-test*). **(H)** Immunohistochemical stainings of teased fiber preparations derived from cultivated sciatic nerve fragments against the myelin protein zero (P0). Arrows point to degraded myelin. Scale bar: 10 μm. **(I)** Immunohistochemical staining of nerve segment cross-sections for CD11b and CD68. Scale bar: 20 μm. The inset shows a costaining of NFH and CD11b. **(J)** Quantification of CD11b and CD68 stainings. Five sections per nerve segment were analyzed; three segments per genotype (*n* = 3, unpaired, two-tailed *t-test*). *p < 0.05; **p < 0.01; ***p < 0.001.

Besides an excessive myelin production, impaired myelin degradation might represent an additional mechanism responsible for causing the myelin infoldings within peripheral nerves of Plekhg5-deficient animals. The previously identified role of Plekhg5 in autophagy regulation (Lüningschrör et al., 2017) prompted us to look for any autophagy defects in peripheral nerves (Figures 3A–C).

First, we analyzed several autophagy markers in sciatic nerve lysates by Western blot (Figures 3A,B). We detected reduced levels of LC3-II, enhanced levels of Lamp1, and unaffected levels of LC-I and p62 (Figures 3A,B). On the cellular level, we frequently detected accumulations of Lamp1^+^ vesicles in sciatic nerve cross-sections (Figure 3C). These findings suggest an accumulation of Lamp1^+^ late endosomes and a reduced number of autophagosomes, which could affect both the degradation of myelin and also the turnover of signaling endosomes. This may lead to the enhanced Erk and Akt signaling.

Next, we wondered whether *Plekhg5*-deficiency results in autophagy impairments within Schwann cells. Therefore, we cultured primary Schwann cells of mutant mice transduced with a lentiviral GFP-RFP-LC3 reporter (Figures 3D,E). Using this reporter, autophagosomes and autolysosomes can be distinguished based on their fluorescent signals as the GFP signal is rapidly lost after fusion of autophagosomes and lysosomes due to the pH drop, whereas the RFP signal persists (Klionsky et al., 2016). Under basal conditions, no difference in the number of autophagosomes was observed in *Plekhg5*-deficient Schwann cells (Figures 3D,E). Upon treatment with Bafilomycin A1, which blocks the fusion of autophagosomes and lysosomes (Klionsky et al., 2016), the increase in the number of autophagosomes in Plekhg5-depleted cells was significantly lower compared to wild-type cells, indicating an impaired autophagosome biogenesis (Figures 3D,E).

Because we did not detect any signs for demyelination within peripheral nerves of Plekhg5-deficient mice, we aimed to promote axon damage and demyelination by an *ex vivo* approach to investigate impairments in myelin breakdown (Fernandez-Valle et al., 1995). As previously described, we cultured segments of the sciatic nerve from *Plekhg5*-deficient mice to simulate nerve injury. After 5 days in culture, we counted the remaining intact myelin sheaths and observed a significantly increased number in segments derived from mutant mice (Figures 3F,G). In teased fiber preparations of cultured nerve segments, we detected myelin clusters positive for P0 in control mice, suggesting a Schwann cell–mediated myelin clearance in autophagosomes as previously described (Gomez-Sanchez et al., 2015). In contrast, such P0-positive clusters were not detectable in Plekhg5-deficient mice (Figure 3H), indicating an impaired sequestering of myelin debris into autophagosomes.

Schwann cell autophagy–mediated myelin degradation was described as the initial response to injury (Gomez-Sanchez et al., 2015). However, after this initial phase, myelin is being degraded *via* phagocytosis mediated by macrophages (Brosius Lutz and Barres, 2014). Thus, we also examined the behavior of macrophages in nerve segments after 7 days in culture. On cross-sections of nerve segments isolated from wild-type mice, we observed a strong immunoreactivity of CD11b and CD68 (Figure 3I). As previously described, after nerve injury (Vega-Avelaira et al., 2009), macrophages frequently clustered around axons in a “ring-like” morphology (Figure 3I, inset). In nerve segments of Plekhg5-deficient mice, we observed a reduced macrophage activity as shown by a smaller area covered by both CD11 and CD68 (Figure 3J).

Taken together, this set of experiments shows that the absence of Plekhg5 results in impaired autophagy in Schwann cells, accumulations of Lamp1^+^ organelles, and defective clearance of myelin in cultured nerve segments.

### Reduced Recruitment of T-Lymphocytes Within the Sciatic Nerve

Previous studies identified Plekhg5 as an activator of the transcription factor NF-κB (Maystadt et al., 2007). Thus, we wondered whether the depletion of Plekhg5 resulted in any transcriptional changes that cause or contribute to the phenotype observed in peripheral nerves of Plekhg5-deficient mice.

**Figure 4:**
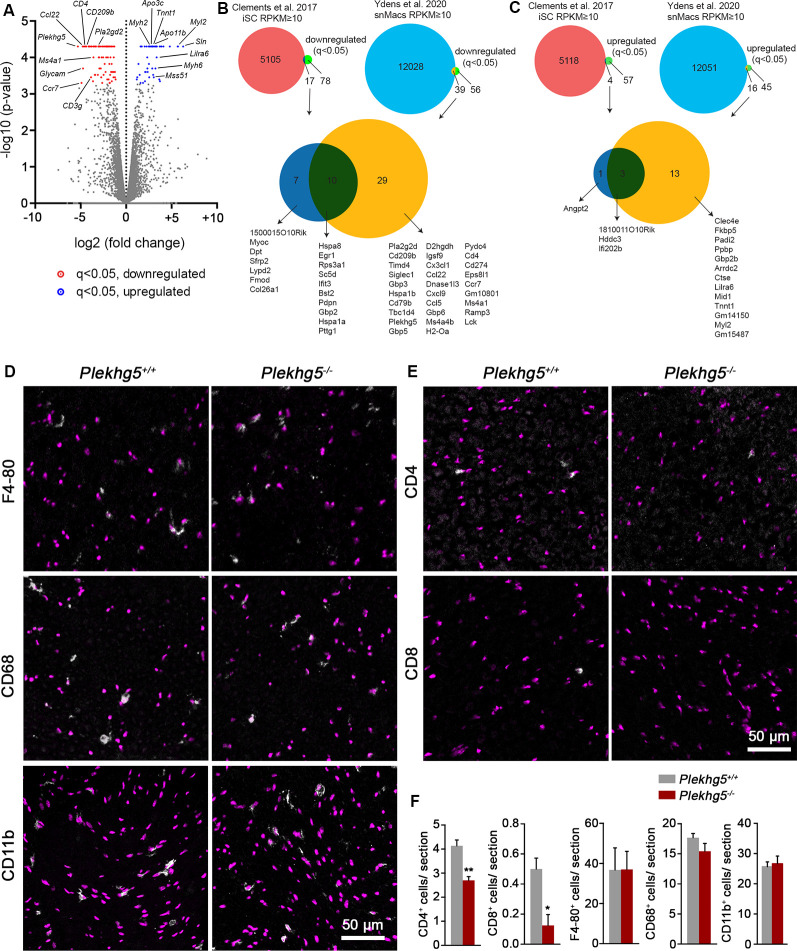
Lack of Plekhg5 results in a reduced immune response within the sciatic nerve. **(A)** Volcano plot showing the significance of transcript change [−log_10_(*p*-value)] vs. the magnitude of change [log_2_(fold change)]. Downregulated transcripts with *q* < 0.05 are marked in red, upregulated transcripts with *q* < 0.05 are marked in blue. Data points for transcripts with log2FC <−10 or >10 (all of which were not significantly altered) were omitted for visualization purposes. Three animals per genotype were analyzed. **(B,C)** Venn diagrams depicting the number of downregulated **(B)** or upregulated **(C)** genes enriched in iSCs and snMac (upper panel). Overlap between the downregulated **(B)** or upregulated **(C)** transcripts found in iSCs and/or snMacs (lower panel). iSCs—isolated Schwann cells. snMacs—sciatic nerve Macrophages. **(D,E)** Immunohistochemical stainings of sciatic nerve cross-sections for the macrophage markers F4–80, CD-68, and CD11b and the T-lymphocyte markers CD4 and CD8. **(F)** For each marker, at least eight sections per animal were analyzed. Three animals per genotype (*n* = 3, unpaired, two-tailed *t-test*). *p < 0.05; **p < 0.01.

For an unbiased approach, we performed RNAseq with RNA derived from the sciatic nerve of 12-month-old animals. In Plekhg5-deficient sciatic nerves, we detected 95 transcripts that were significantly (*q* < 0.05) downregulated relative to wild-type controls and a lower number of 61 transcripts that were upregulated (Figure 4A; Supplementary Table S2). To assign the dysregulated transcripts to specific cell types, we used published data sets to investigate their enrichment in isolated Schwann cells (iSCs) and/or sciatic nerve macrophages (snMacs; Figures 4B,C; Clements et al., 2017; Ydens et al., 2020). Of the 95 downregulated transcripts, only seven were enriched in iSCs, and 29 of them overlapped with transcripts enriched in snMacs. Ten transcripts were found in both cell types. The downregulated iSC transcripts were mostly associated with the assembly of the extracellular matrix (ECM; *Col26a1*, *Fmod*, *Dpt*, *MyoC*). The downregulated transcripts enriched in snMac were associated with the interferon response (*Gpb3, Gpb5, Gpb6)*, chemokines (*Ccl22, Ccl5, Cxcl9, Cx3cl1*), and cell surface proteins (*CD209b, H2-Oa, Siglec1, Timd4, Ms4a1*; Supplementary Figure S1B). Among them, we also detected the transcript encoding for *Pla2g2d*, encoding an extracellular phospholipase, secreted by macrophages for myelin breakdown (Martini et al., 2008). Among the downregulated transcripts, which were not expressed in iSC or snMac, we detected a reduced expression of T-cell–associated transcripts (*CD3e, CD3g, CD4, Lck*). Overall, the downregulated genes were enriched for validated target genes of the transcription factor NF-κB (Supplementary Figure S1C).

Of the 61 transcripts upregulated in Plekhg5-deficient sciatic nerves, 13 transcripts showed an overlap with transcripts enriched in snMacs, and only one of the upregulated transcripts (*Angpt2*) could be identified as iSC-enriched. Three transcripts were detected in both cell types (Figure 4C). The upregulated transcripts, which could not be assigned to iSCs or snMacs, appeared to be predominantly muscle-enriched transcripts (*Sln, Mss51, Myh2, Myh6, Myh7*; Supplementary Figure S1A).

In addition, several of the up- and downregulated transcripts overlapped with transcriptional changes upon nerve injury (Arthur-Farraj et al., 2017; Supplementary Figures S1D–G). Notably, we detected Plekhg5 among the downregulated genes after nerve injury.

Taken together, a greater proportion of the dysregulated transcripts could be assigned to snMacs than to iSCs, suggesting that, at least on the transcriptional level, Plekhg5 depletion has a higher impact on macrophages than on Schwann cells.

To address whether this signature is caused by an altered number of macrophages, we quantified the number of F4/80-, CD11b-, and CD68-positive cells in sciatic nerve cross-sections (Figures 4D,F; Supplementary Figure S2). Because we also found a downregulation of several T-cell–attracting chemokines and T-cell markers, we examined the number of CD4- and CD8-positive cells in addition (Figures 4E,F). We found an unaltered number of macrophages in sciatic nerve from Plekhg5-deficient mice but a reduced number of both CD4- and CD8-positive cells (Figures 4E,F; Supplementary Figure S2). In line with reduced macrophage activity in cultured nerve segments upon Plekhg5 depletion, these results indicate a macrophage dysfunction, which lead to a reduced T-cell infiltration into the nerve.

## Discussion

Proper myelination requires balanced Erk1/2 and Akt signaling in Schwann cells. Hyperactivation of the PI3 kinase pathway in mice with a targeted disruption of Pten in Schwann cells resulted in increased levels of Akt signaling, leading to focal hypermyelination, myelin outfoldings, and tomacula in peripheral nerves (Goebbels et al., 2012). In contrast, hyperactivation of Erk1/2 signaling by an inducible Raf-kinase transgene in myelinated Schwann cells resulted in Schwann cell dedifferentiation and severe demyelination in the absence of axonal damage (Napoli et al., 2012).

An enhanced signaling by Erk1/2 has been reported in several mouse models for HMSN with myelin abnormalities, such as myelin infoldings, outfoldings, or tomacula (Fischer et al., 2008; Nadra et al., 2008; Lee et al., 2012, 2013). Of particular interest is the CMT1C model due to expression of the human mutant SIMPLE protein. CMT1C mice display a similar phenotype to peripheral nerves from Plekhg5-deficient mice with infolding of the myelin sheath (Lee et al., 2013). SIMPLE recruits components of the ESCRT machinery to endosomal membranes regulating endosome-to-lysosome delivery (Lee et al., 2012). Endosome-to-lysosome trafficking of cell surface receptors is a major mechanism to regulate the intensity and duration of signal transduction (Miaczynska et al., 2004). When cell surface receptors are internalized upon ligand binding and accumulate due to impaired trafficking or degradation while the signaling capability persists, the overall intensity of downstream signal transduction is enhanced. Such an effect has been observed upon depletion of SIMPLE in Schwann cells, which causes an impaired delivery of ErbB3-endosomes to lysosomes. These defects in ligand-induced receptor degradation result in prolonged Erk1/2 activation (Lee et al., 2012). In this context, it is tempting to speculate that the increased levels of phosphorylated Akt and Erk in sciatic nerves of Plekhg5-deficient mice are caused by an impaired turnover and/or trafficking of signaling endosomes. This hypothesis is supported by the accumulation of Lamp1^+^ vesicles in the sciatic nerves of Plekh5-deficient mice, which suggests an impaired clearing of organelles from the endosomal pathway. Such a scenario is in agreement with our previous study identifying Plekhg5 as a regulator of synaptic vesicle turnover (Lüningschrör et al., 2017). Nevertheless, more experimental work is necessary to validate this hypothesis.

Our data suggest that the impaired autophagy also affects the myelin degradation as an additional mechanism contributing to the infolding of the myelin sheath. We observed reduced LC3-II levels in sciatic nerve lysates of Plekhg5-deficient mice and a reduced number of autophagosomes in cultured Schwann cells. In addition, we detected an enrichment of Lamp1 in sciatic nerve lysates and accumulation of Lamp1^+^ vesicles in sciatic nerve cross-sections. In cultured nerve segments, loss of Plekhg5 resulted in impaired degradation of myelinated axons, supporting the idea of a defective myelin turnover. In contrast to the accumulation of Lamp^+^ organelles, we found no enrichment of p62, suggesting that there is no general proteostasis impairment in Plekhg5-deficient sciatic nerves. These findings are in agreement with previous reported results showing that Plekhg5 affects synaptic vesicle turnover in motoneurons without disturbing general proteostasis (Lüningschrör et al., 2017).

Schwann cell autophagy–mediated myelin degradation was described as the initial response to injury (Gomez-Sanchez et al., 2015). However, after this initial phase, myelin is being degraded *via* phagocytosis mediated by macrophages (Brosius Lutz and Barres, 2014). To initiate myelin clearance by macrophages, Schwann cells reportedly have the capability to act as facultative antigen-presenting cells, expressing MHC complex II molecules (Bergsteinsdottir et al., 1992; Meyer zu Hörste et al., 2010). In addition, several myelin proteins were described as autoantigens in neuropathologies (Braun et al., 1982; de Rosbo and Ben-Nun, 1998; Kaushansky et al., 2010). Antigens presented by Schwann cells are recognized by T-lymphocytes to initiate macrophage-mediated degradation. In mouse models of demyelinating PNS disorders, both T-lymphocytes and macrophages play a crucial role in myelin breakdown (Wang Ip et al., 2006). In mice heterozygously deficient in P0, an animal model for a subtype of hereditary neuropathy, T-lymphocytes are present in the demyelinating nerves (Shy et al., 1997). Correlating with age-dependent neuropathy in *P0*^+/−^ mice, the infiltration of both cell types increases with age (Schmid et al., 2000; Carenini et al., 2001). In line with these observations, depletion of T-cells in *P0*^+/−^ mice leads to improved myelin maintenance (Schmid et al., 2000). Furthermore, *P0*^+/−^ mice deficient for the macrophage colony factor display a reduced number of macrophages in peripheral nerves and a less severe demyelination (Carenini et al., 2001). Similar to *P0*^+/−^ mice, we detected an approximate 50% reduction of P0 in Plekhg5-deficient mice. Despite this reduction, which apparently triggers an immune response in *P0*^+/−^, no signs of immune cell infiltration was observed upon depletion of Plekhg5, even in 24-month-old animals. Consistently, we detceted no sign for a demyelination in Plekhg5-deficient mice.

Using RNA-seq, we identified the downregulation of several macrophage-enriched transcripts. Among these, we detected T-cell–attracting chemokines and also a number of cell surface proteins such as Siglec-1, involved in T-cell interaction or pathogen recognition. Notably, depletion of Siglec-1 in P0^−/−^ mice results in an attenuated demyelination and a reduced number of CD8^+^ T-lymphocytes in peripheral nerves (Kobsar et al., 2006). We also detected a small number of Schwann cell transcripts involved in ECM assembly. Previous studies show that the ECM plays an important role not only for Schwann cell function, including myelination, but also for macrophage recruitment (Chen et al., 2015a,b). In summary, the signature of transcriptional changes observed here suggests a defective interplay between multiple cell types involving the impaired activation of macrophages, which results in an improper detection of defective myelin and consequently in a reduced recruitment of T-cells. Interestingly, we did not detect an upregulation of the Schwann cell–derived chemokine Ccl2, which is involved in macrophage activation in *P0*^+/−^ mice (Fischer et al., 2008). Elevated levels of Erk1/2 signaling in *P0*^+/−^ mice mediate the increased expression of Ccl2. Similar to *P0*^+/Ȓ^ mice, we also detected elevated levels of Erk1/2 in Plekhg5-deficient mice but no alterations in the level of Ccl2, suggesting an impaired transcriptional response downstream of Erk1/2 signaling. Ccl2 is a well-established target of the transcription factor NF-kB (Ueda et al., 1994, 1997). In this context, it is of particular interest that Plekhg5 is an activator of transcription factor NF-kB (Maystadt et al., 2007), which might represent the missing link between the alteration in myelination and an enhanced Erk1/2 signaling, but without triggering the immune response described in *P0*^+/−^ mice and other models for demyelinating PNS disorders (Wang Ip et al., 2006).

Overall, the downregulated genes upon Plekhg5 depletion were enriched for targets of NF-kB. Although we did not experimentally investigate the NF-kB signaling pathway, it is tempting to speculate that Plekhg5 depletion results in a reduced signaling by NF-kB, leading to an impaired macrophage activation, which causes a decreased expression of chemokines for T-cell attraction.

In contrast to the downregulated genes, we can only speculate on the signature of the upregulated genes. The only upregulated iSC transcript is *Angpt2*, encoding a ligand for TIE2, a receptor involved in regulating angiogenesis (Jeltsch et al., 2013). The majority of the upregulated genes are expressed in neither iSCs nor in snMac. These transcripts were, rather, myosins (*Myl2, Myh6, Myh5, Myh7*) or genes encoding for proteins associated with muscle cell function (*Egln3, Lmod2, Myoz, Ankrd2, Mss51, Sln*). Such a signature might be related to transcriptional alteration in endothelial cells, which line the interior surface of blood vessels within the nerve. The actomyosin contractility of these cells determines the microvascular permeability of blood vessels by regulating cell junctions. Strikingly, Plekhg5 has an established role in cell junction regulation of endothelial cells (Ngok et al., 2012). Furthermore, hyperactivation of Erk1/2 in myelinated Schwann cells resulted in breakdown in the blood–brain barrier (Napoli et al., 2012). However, further studies are needed to experimentally test this hypothesis within the nervous system.

In conclusion, our data add further weight to the notion that the interaction between the immune system and the nervous system needs to be tightly regulated and that a dysregulation of this interplay contributes to the axon pathology in demyelinating PNS disorders and motoneuron disease.

## Data Availability Statement

The datasets generated for this study can be found in the NCBI’s Gene Expression Omnibus, GEO Series accession number: GSE127319.

## Ethics Statement

The animal study was reviewed and approved by the Landesumweltamt (LANUV), Recklinghausen, NRW. Written informed consent was obtained by the owners for the participation of their animals in the study.

## Author Contributions

CS and PL designed and performed experiments, analyzed data, and wrote the manuscript. UW and BM performed experiments. PH designed and performed experiments. MB and SA analyzed data. MS provided critical comments and resources. BK and CK designed experiments, provided resources, and supervised the study.

## Conflict of Interest

The authors declare that the research was conducted in the absence of any commercial or financial relationships that could be construed as a potential conflict of interest.

## Footnotes

^1^http://www.bioinformatics.babraham.ac.uk/projects/trim_galore/

^2^https://cutadapt.readthedocs.io/en/stable

^3^https://github.com/alexdobin/STAR

^4^http://cufflinks.cbcb.umd.edu/

^5^https://david.ncifcrf.gov
