# Supplementary material for: Absence of Plekhg5 Results in Myelin Infoldings Corresponding to an Impaired Schwann Cell Autophagy, and a Reduced T-Cell Infiltration Into Peripheral Nerves
Source: Front Cell Neurosci. 2020 Jul 7;14:185. doi: 10.3389/fncel.2020.00185 (PMC7358705; doi:10.3389/fncel.2020.00185)
Supplement: Supplementary file 2 [file Data_Sheet_1.PDF]

Supplementary Information

Absence of Plekhg5 results in myelin infoldings corresponding to an impaired Schwann cell autophagy, and a reduced T-cell infiltration into peripheral nerves

Patrick Lüningschrör[1+*], Carsten Slotta[2,3+], Peter Heimann[2], Ulrich M. Weikert[2], Michael Briese[1], Silke Appenzeller[4,5], Bita Massih[1], Michael Sendtner[1], Christian Kaltschmidt[2+], Barbara Kaltschmidt[2,3+*]

[1] Institute of Clinical Neurobiology, University Hospital Wuerzburg, 97078 Wuerzburg, Germany
[2] Department of Cell Biology, University of Bielefeld, Universitaetsstr. 25, 33501 Bielefeld, Germany
[3] Molecular Neurobiology, University of Bielefeld, Universitaetsstr. 25, 33501 Bielefeld, Germany
[4] Core Unit Systems Medicine, University of Wuerzburg, 97080 Wuerzburg, Germany
[5] Comprehensive Cancer Center Mainfranken, University Hospital Wuerzburg, 97080 Wuerzburg, Germany

+ These authors contributed equally to this work

* Corresponding Authors:

Barbara Kaltschmidt, Molecular Neurobiology, Department of Cell Biology, University of Bielefeld, Universitaetsstr. 25, 33501 Bielefeld, Germany,
Phone.: + 49 521 106 5624; Fax: + 49 521 106 5654; E-Mail: b.kaltschmidt@uni-bielefeld.de

Patrick Lüningschrör, Institute of Clinical Neurobiology, University Hospital Wuerzburg, 97078 Wuerzburg, Germany
Phone.: +49 931 201 44030; E-Mail: Lueningsch_P@ukw.

Supplementary Figure 1

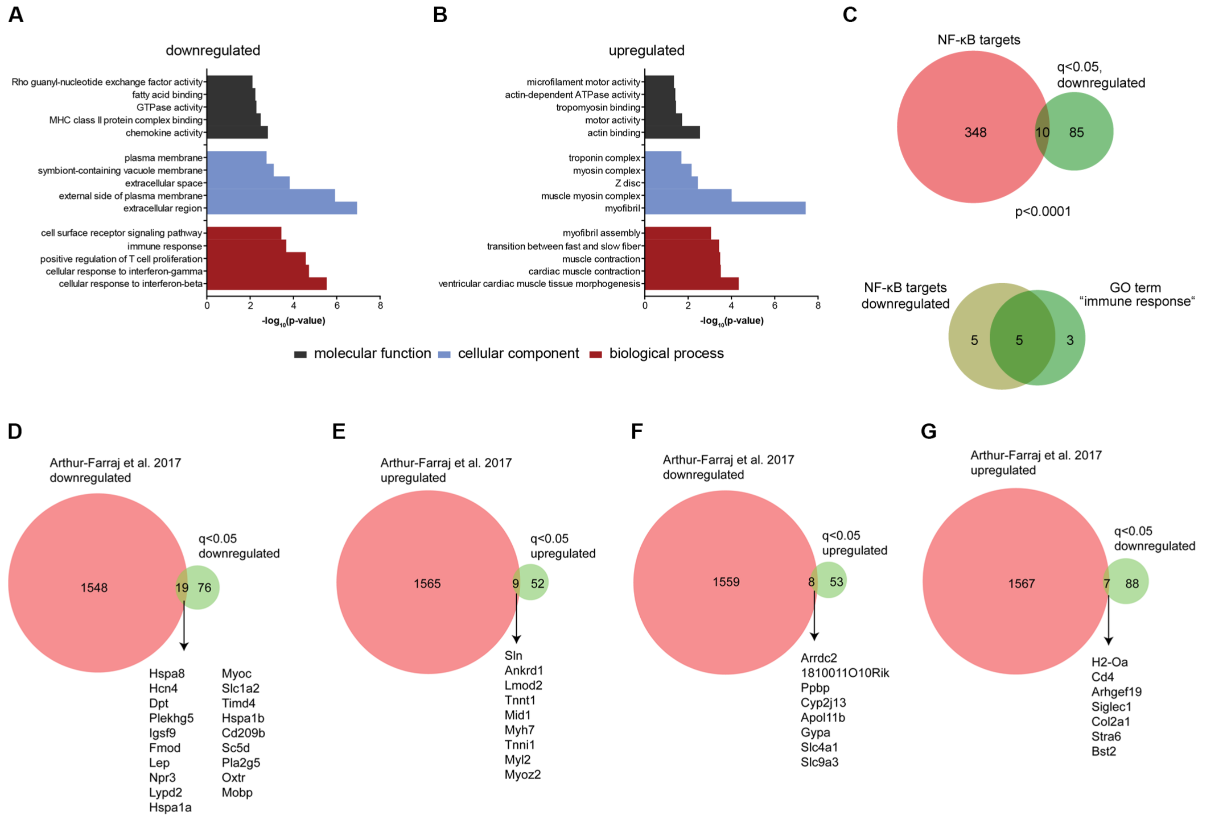

A, B Gene-ontology (GO) term analysis of significantly downregulated (**A**) and upregulated genes (**B**).

C Venn diagrams depicting the number of NF-κB targets within all downregulated genes (upper diagram) and within the GO term "immune response" (lower diagram).

D Venn diagram depicting the overlap between the downregulated transcripts upon Plekhg5 depletion and the downregulated transcripts upon nerve injury.

E Venn diagram depicting the overlap between the upregulated transcripts upon Plekhg5 depletion and the upregulated transcripts upon nerve injury.

F Venn diagram depicting the overlap between the upregulated transcripts upon Plekhg5 depletion and the downregulated transcripts upon nerve injury.

G Venn diagram depicting the overlap between the downregulated transcripts upon Plekhg5 depletion and the downregulated transcripts upon nerve injury.

Supplementary Figure 2

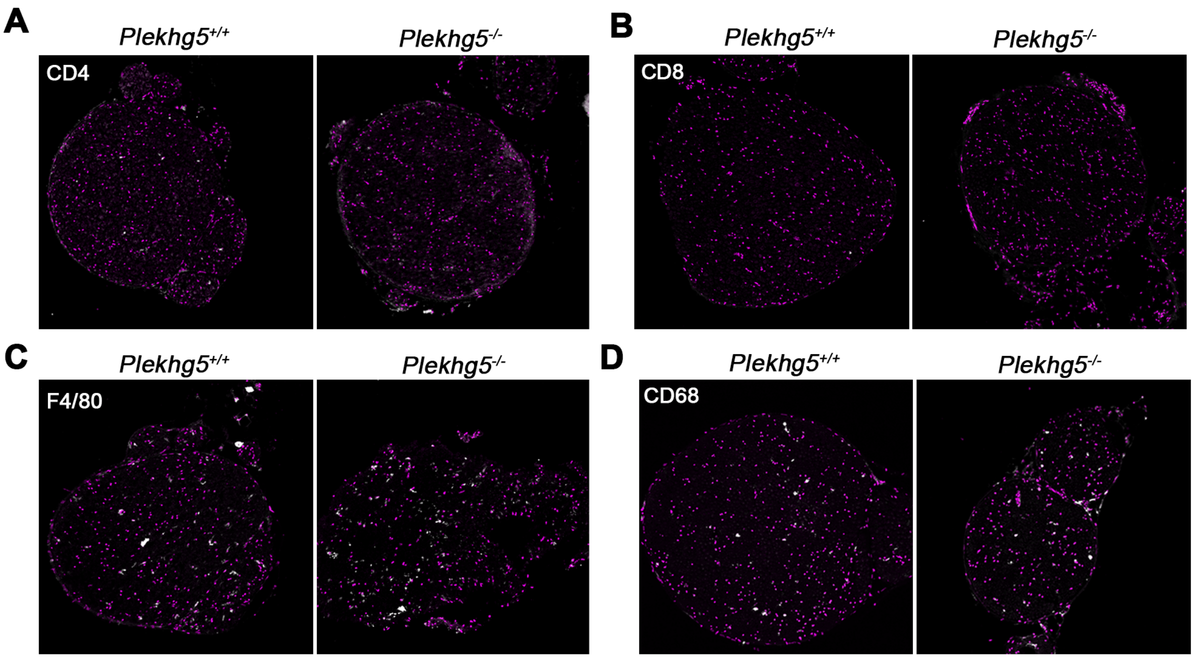

A – D Overview images of the immunohistochemical stainings shown in Figure 4. Sciatic nerve cross sections were stained for the T-lymphocyte markers CD4 (**A**) and CD8 (**B**), and the macrophage markers F4-80 (**C**) and CD-68 (**D**).

Supplementary Table 1

List of Antibodies

Western Blot

anti-Lamp1	DSHB (1D4B)
anti-p62	Progen (GP62-C)
anti-LC3	Novus Biologicals (NB100-2220)
anti-Calnexin	Sicgen (AB0037-200)
anti-Tuj1	Neurogenomics (MO15013)
anti-phospho Akt Ser647	Cell Signaling, D9E, #4060
anti-Akt	Cell Signaling, #9272
anti-phospho- p42/p44 MAPK (Erk1/2)	Cell Signaling, #9106
anti- p42/p44 MAPK (Erk1/2)	Cell Signaling, L34F12, #4696
anti-MPZ, P0	Proteintech, 10572-1-AP
anti-PLP1	Novus Biologicals NBP1-87781
anti-PMP22	Santa Cruz, G-6, sc-515199

Immunohistochemisrty

anti-CD4	Serotec (YTS191.1)
anti-CD8	Serotec (YTS169.4)
anti-CD68	Serotec (CI:A3-1)
anti-F4/80	Serotec (FA-11)
anti-MPZ	Abcam (Ab39375)
anti-CD11b	Bio-Rad (M1/70.15)
anti-Lamp1	DSHB (1D4B)

List of Oligonucleotides

Ppia-Fwd	5‘-GTCTCCTTCGAGCTGTTTGC-3‘
Ppia-Rev	5‘-GTCTCCTTCGAGCTGTTTGC-3‘
EeF2-Fwd	5‘-GGAACATGTCAGTCATCGCC-3‘
EeF2-Rev	5‘-GGAACATGTCAGTCATCGCC-3‘
Plp1-Fwd	5‘-CTCCAAAAACTACCAGGACTATGAG-3‘
Plp1-Rev	5‘-AGGGCCCCATAAAGGAAGA-3‘
Mag-Fwd	5‘-TGATAAGTATGAGTCCAGAGAGGTC-3‘
Mag-Rev	5‘-TGATAAGTATGAGTCCAGAGAGGTC-3‘
Pmp22-Fwd	5’-AATGTTGATCATGCCATCTCCC-3‘
Pmp22-Rev	5’-CGGTGCCTGTTTCAGTTCAA-3’
P0-Fwd	5’-AATGTTGATCATGCCATCTCCC-3‘
P0-Rev	5‘-TTCTTTGGTGCTCTCTCCAGC-3‘
